# The Linker Histone H1.2 Is an Intermediate in the Apoptotic Response to Cytokine Deprivation in T-Effectors

**DOI:** 10.1155/2014/674753

**Published:** 2014-02-13

**Authors:** Megha Garg, Lakshmi R. Perumalsamy, G. V. Shivashankar, Apurva Sarin

**Affiliations:** ^1^National Centre for Biological Sciences, Bellary Road, Bangalore, Karnataka 560065, India; ^2^Department of Biotechnology, Mysore University, Mysore, Karnataka 570005, India; ^3^Department of Biotechnology, Indian Institute of Technology Madras, Chennai, Tamil Nadu 600036, India; ^4^Mechanobiology Institute, National University of Singapore, Singapore 117411

## Abstract

Tissue homeostasis is a dynamic process involving proliferation and the removal of redundant or damaged cells. This is exemplified in the coordinated deletion—triggered by limiting trophic factors/cytokines in the extracellular milieu—of differentiated T cells overproduced during the mammalian immune response. However, mechanisms by which extracellular cues are perceived and transduced as apoptotic triggers remain incompletely understood. T-effectors are dependent on cytokines for survival and undergo apoptosis following cytokine withdrawal. Here we report that leptomycin B (LMB), an inhibitor of nuclear export machinery, protected T-effectors from apoptosis implicating a nuclear intermediate in the apoptotic pathway. Evidence is presented that the linker histone H1.2 localizes to the cytoplasm, by a mechanism sensitive to regulation by LMB, to activate apoptotic signaling culminating in nuclear and mitochondrial damage in T-effectors in response to cytokine deprivation. H1.2 is detected in a complex with the proapoptotic mitochondrial resident Bak and its subcellular localization regulated by Jun-N-terminal kinase (JNK), an intermediate in the apoptotic cascade in T-effectors. These data suggest that metabolic stressors may impinge on H1.2 dynamics favoring its activity at the mitochondrion, thereby functioning as a molecular switch for T-effector apoptosis.

## 1. Introduction

Cells divide and differentiate to acquire distinct cell fates in multicellular organisms. However, differentiation and proliferation are balanced by the programmed deletion of excess or damaged cells, which is critical for tissue homeostasis. Core elements of the cellular machinery regulating cell death are conserved in different phyla [1] and at least two major pathways regulating apoptosis have been described in mammalian cells. Signaling cascades initiated by ligand-receptor engagements of the TNFR (tumor necrosis factor receptor) superfamily constitute the extrinsic pathway [2]. The intrinsic pathway is activated by metabolic and genotoxic stressors and integrated at the mitochondrion, with members of the Bcl-2 family, which comprise both pro- and antiapoptotic members emerging as dominant regulators of these responses [1, 3, 4]. Cross talk between these two pathways is well documented. Cell-death pathways are variously deployed during development and in adult tissues as seen in the nervous, reproductive, and immune system where cells are overproduced and then removed by apoptosis [5–9]. Competition for limiting nutrients in the extracellular milieu is a widely prevalent mechanism for the regulation of cell number [10–12]. However, mechanisms by which cells perceive changes in their microenvironment are not fully understood.

In this study, we sought to address this question in a well-characterized model system that recapitulates the deletion of differentiated T cells in cell culture. The mature T cell compartment is characterized by dramatic increase in cell number in response to antigen challenge, followed by the eventual elimination of antigen-reactive T-effector cells so generated, a cycle repeated throughout adult life and necessary for immune functionality [13, 14]. T-effectors generated in the immune response depend on cytokines or trophic factors for nutrient uptake and experiments *in vitro* and *in vivo* model systems indicate that the apoptotic response to nutrient deprivation is mediated by reactive oxygen species- (ROS-) dependent signaling that converges on the mitochondrion [15–18]. While T-cell dependence on cytokines and the ROS-Bcl-2 family signaling axis for survival and apoptosis, respectively, is well established, the cellular machinery that senses and integrates changes in the extracellular milieu remains uncharacterized and forms the focus of this study.

A critical role for nuclear effectors in initiating apoptotic signaling in T-effectors was indicated in our experiments since blocking nuclear export machinery protected cells from apoptosis. Building on a previously described role for the linker histone H1.2 in propagation of apoptotic cascades we followed the spatial dynamics of the linker histone H1.2 isoform in T-effectors. Our experiments show that the nuclear export machinery and JNK activity regulate H1.2 translocation and activity in T-effectors undergoing apoptosis. We provide evidence of H1.2 interaction with the Bcl-2 family proapoptotic protein Bak and propose that H1.2 converges on apoptotic signaling cascades integrated at the mitochondrion. Its role in apoptosis is indicated by the abrogation of apoptotic damage in response to cytokine deprivation following H1.2 ablation in T-effectors. Finally, our experiments implicate the Jun-N-terminal kinase (JNK) in regulating H1.2 dynamics, suggesting a more expansive role for this signaling cascade in tissue homeostasis.

## 2. Materials and Methods

### 2.1. Mice

C57BL/6 mice were obtained from The Jackson Laboratory (Maine, USA) and maintained at the Animal Facility at the National Centre for Biological Sciences (NCBS), Bangalore, India. Manipulations involving animals were approved by the Institutional Animal Ethics Committees of NCBS, Bangalore, India, and followed norms specified by the Committee for the Purpose of Control and Supervision of Experiments on Animals (CPCSEA), Government of India.

### 2.2. Reagents

Antibodies were used at dilutions indicated and procured from the following sources: H1 (1 : 1000) and p38MAPK (1 : 1000) from Santa Cruz (Santa Cruz, CA); AIF (1 : 500) from Chemicon (Billerica, MA); H1.2 (1 : 1000) from Proteintech Europe (Manchester, UK); H3 (1 : 500), H3Ac (1 : 500), and HP1*α* (1 : 1000) from Upstate Biotechnology (Lake Placid, NY); *α*-actin (1 : 500) and *α*-tubulin (1 : 250) from Neomarker (Fremont, CA); and Bak (1 : 1000), Cox-IV (1 : 250), and p-JNK/SAPK (1 : 500) from Cell Signaling Technology (Beverly, MA). The shRNA plasmids to H1.2 and the scrambled control were from Origene Technologies (Rockville, MD). TMRM (tetramethyl rhodamine methyl ester) was obtained from Sigma (St. Louis); Annexin-V AlexaFluor-488 was from Invitrogen (Carlsbad, USA); anacardic acid, leptomycin B, and SP600125 were from Calbiochem (San Diego, CA).

### 2.3. Generation of T-Effectors

T-cells were isolated from spleens of 6–8-week-old C57BL/6 mice, using mouse IgG coated magnetic (GAMT) beads (NEB, USA) or using CD3+ T cell isolation kits (R&D Systems). T-effectors were generated as described [17]. Briefly, T-cells were stimulated using *α*-CD3/*α*-CD28 bound beads (Invitrogen) at 2.0 × 10^6^ cells/mL or in some instances soluble anti-CD3 (500 ng/mL, clone 2C11; R&D Systems) at 2.5 × 10^6^ T cells/mL in cultures spiked with 10^4^ splenic adherent cells. T-effectors generated after 48 hours were used for assays or continued in culture with 1 *μ*g/mL cytokine interleukin-2 (R&D Systems) for 24–36 hours. Activation was established by expression of CD69 and CD25 along with the changes in morphology and increased size of T-cells [19].

### 2.4. Assays of Apoptosis

Assays of cytokine deprivation were performed as described [17]. 0.4 × 10^6^/mL T-effectors were washed thrice in excess medium and phosphate buffered saline (PBS) to remove bound cytokine and then continued in culture with or without IL-7. Apoptosis was assayed at different time points after cytokine deprivation as indicated in the experiments. For assays of subcellular fractionation, cells were typically harvested at 6 hours for the analysis of H1.2 dynamics and at a delayed time point of 8 hours to examine the translocation of AIF. In experiments where H1.2 and AIF translocations were examined together, samples were harvested at the earlier time point of 6 hours.

To assess apoptotic nuclear damage, cells were stained for 3 minutes with the Hoechst-33342 (1 *μ*g/mL) at ambient temperature and nuclear morphology scored in a minimum of 200 cells in each sample, using a fluorescence microscope equipped with a UV filter. For staining with Annexin-V, 0.3 × 10^6^ T-effectors were incubated with Annexin-V (1 : 60/10^6^ cells) for 15 min at room temperature, washed once with PBS, and analyzed immediately by flow cytometry following the addition of propidium iodide. For the assessment of mitochondrial transmembrane potential, cells were incubated with a 50 nM mitochondria-specific dye, TMRM, in medium for 15 minutes at 37°C, followed by two gentle washes with PBS and immediately analyzed by flow cytometry.

### 2.5. Retroviral Infections

For packaging murine retroviruses with shRNA plasmids, 1 *μ*g of DNA was cotransfected with 1 *μ*g of pClEco (packaging vector) in HEK293T cells. After 48 hours, the culture supernatant was concentrated and used for infecting T-cells by previously described protocols [17, 20]. Infected T cells were maintained in RPMI complete medium supplemented with 1 *μ*g/mL IL-2, 2 ng/mL IL-7, and 1 *μ*g/mL puromycin for 3 days. Live cells were isolated using Ficoll and continued in culture with IL-2 for 24 hours before being used in cytokine deprivation assays. Protein levels were assessed by Western blot analysis.

### 2.6. Western Blot Analysis

0.3–0.5 × 10^6^ cells were lysed in a SDS lysis buffer (2% SDS, glycerol, bromophenol blue), 1 M DTT, and 1 M Tris—HCl pH 6.8 supplemented with a protease inhibitor cocktail—aprotinin, leupeptin, and pepstatin (2 *μ*g/mL each), 1 mM PMSF, 1 mM NaF, and 1 mM Na_3_VO_4_ for 10 minutes at 100°C. Using previously established protocols [17, 20], whole cell lysates were resolved by SDS-PAGE and transferred to nitrocellulose membrane (GE Healthcare) and incubated overnight at 4°C with primary antibodies at concentrations recommended by the manufacturers. Membrane was washed thrice with TBS-Tween20 followed by HRP-conjugated secondary antibody (CST, 1 : 1000 dilution) for 1 hour at RT. Membranes were developed either by exposing to X-ray film using an Amersham Hyperprocessor or an ImageQuant LAS 4000 Biomolecular Imager (GE Healthcare).

### 2.7. Subcellular Fractionation

10 × 10^6^ T-effectors were used for the subcellular fractionation protocol [21]. Cytoplasmic and nuclear fractions were obtained using the NE-PER nuclear and cytoplasmic extraction kit (Thermo Scientific) following manufacturer's instructions. Equivalent volumes of nuclear and cytoplasmic fractions were boiled in SDS lysis buffer for Western blot analysis.

### 2.8. Immunoprecipitation

10 × 10^6^ T-effectors cultured in the absence of cytokine were lysed for 1 hour at 4°C in 1% CHAPS lysis buffer supplemented with protease inhibitors (2 *μ*g/mL aprotinin, leupeptin, pepstatin, 1 mM PMSF, 1 mM NaF, and 1 mM Na_3_VO_4_). 10 *μ*L antibody was used for precipitating immune complex for 2 hours at 4°C using protein G agarose beads (Pierce Biotechnology) on a rotator. Beads were washed five times with ice-cold PBS at 1700 rpm and then boiled in SDS lysis buffer for 10 min for the Western blot analysis. When the cytoplasmic fraction was used as an input for the IP, 20 × 10^6^ T-cells were subjected to subcellular fractionation as described above and the cytoplasmic fraction obtained was used for immunoprecipitation. True-blot HRP (Ebioscience) has been used for immunoblots of Bak and H1.2.

### 2.9. Statistical Methods

All graphs show data presented as mean ± SD from a minimum of three independent experiments. Statistical significance was calculated using the two population Student's *t*-tests, with the following confidence intervals: *99% and **99.9%.

## 3. Results

### 3.1. Apoptosis in T-Effectors Is Regulated by Nuclear Events

**Figure 1:**
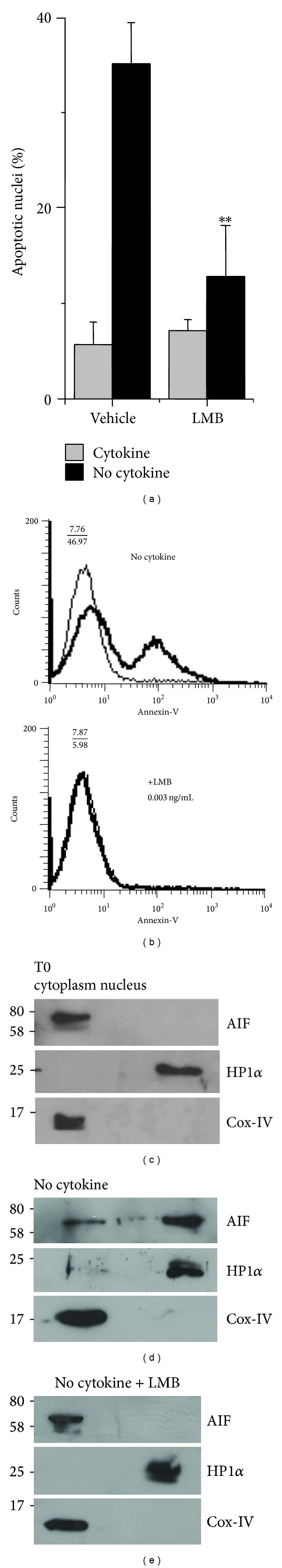
LMB blocks cytokine deprivation induced apoptotic damage in T-effectors. (a)-(b) 0.3 × 10^6^ T-effectors were cultured with or without IL-7 (20 ng/ml) with the addition of vehicle control or LMB (0.003 ng/ml). Apoptotic nuclear damage (a) and Annexin-V binding (b) were scored after 8 and 15 hours, respectively. The dotted lines in (b) indicate cells continued in cytokine. The graph shows mean ± SD from 4 experiments. **P < 0.001. (c)–(e) 10 × 10^6^ T-effectors were cultured in the indicated conditions (T0: freshly activated) and 8 hours later cells were fractionated as described in Materials and Methods. Immunoblots of nuclear and cytoplasmic fractions obtained were probed for AIF, HP1*α*, and Cox-IV. Data are representative of three independent trials.

T-cells proliferate and differentiate in response to antigen, to generate lineage-committed effectors, the bulk of which die, marking termination of the immune response [14]. Key elements of this process can be recapitulated *in vitro*, permitting investigations into the molecular regulation of T-cell apoptosis [16–18, 20, 21]. Using this experimental system we show that cytokine withdrawal from T-effectors triggers apoptotic damage characterized by nuclear fragmentation and externalization of phosphatidylserine (PS) at the cell membrane (Figures 1(a) and 1(b)). Loss of mitochondrial integrity is another feature of cells undergoing apoptotic duress and is reflected in the spatial redistribution of the flavoprotein, AIF (apoptosis inducing factor) [22]. AIF is a mitochondrial intermembrane space resident protein, which is released from mitochondria as a consequence of loss of outer mitochondrial membrane integrity and translocates to the nucleus to mediate DNA damage [21, 23]. In T-cells cultured in cytokine assessed prior to the onset of the deprivation protocol (T0), AIF is detected in the cytoplasmic fraction consistent with its localization to mitochondria (Figure 1(c)). Subcellular fractionation of T-cells following cytokine withdrawal indicated that a substantial signal for AIF is detected in the nuclear fraction (Figure 1(d)). In these assays, we also probed for the distribution of the nuclear heterochromatin binding protein Hp1*α* and the mitochondrial matrix resident Cox-IV to establish purity of the fractions (Figures 1(c)–1(e)).

Leptomycin B (LMB), a Crm1 inhibitor that blocks nuclear export, substantially reduced apoptotic damage in T-effectors (Figures 1(a) and 1(b)), implicating a nuclear intermediate in the cascade. LMB is typically used at concentrations that range from 0.3 to 10 ng/mL although lower concentrations are also effective at blocking nuclear export [24]. Since 0.3 ng/mL LMB was toxic to T-cells in culture, we tested lower concentrations and found that 0.03 mg/mL or 0.003 ng/mL LMB was well tolerated by T-effectors cultured with cytokine. Since both concentrations also protected from apoptosis with comparable efficacy (not shown), the experiments using the lower concentrations of LMB have been used in the current study. The distribution of AIF in cells cultured without cytokine but in the presence of LMB (Figure 1(e)) was restored to the pattern of live cells (Figure 1(c)), indicating that LMB prevented damage to the mitochondrial outer membrane, a necessary step for the release of AIF. These observations suggested that events in the nucleus influence early, premitochondrial steps of the apoptotic cascade. The linker histone H1.2 has been shown to initiate apoptotic cascades integrated by the mitochondrion, first reported in the context of the DNA damage response in immature T-cells [25]. Activation of H1.2 dependent apoptotic signaling is associated with its displacement from the nucleus. Hence, in subsequent experiments we assessed the cellular distribution and possible involvement of H1.2 in T-effector apoptosis.

### 3.2. Changes in H1.2 Localization in T-Effectors Undergoing Apoptosis

**Figure 2:**
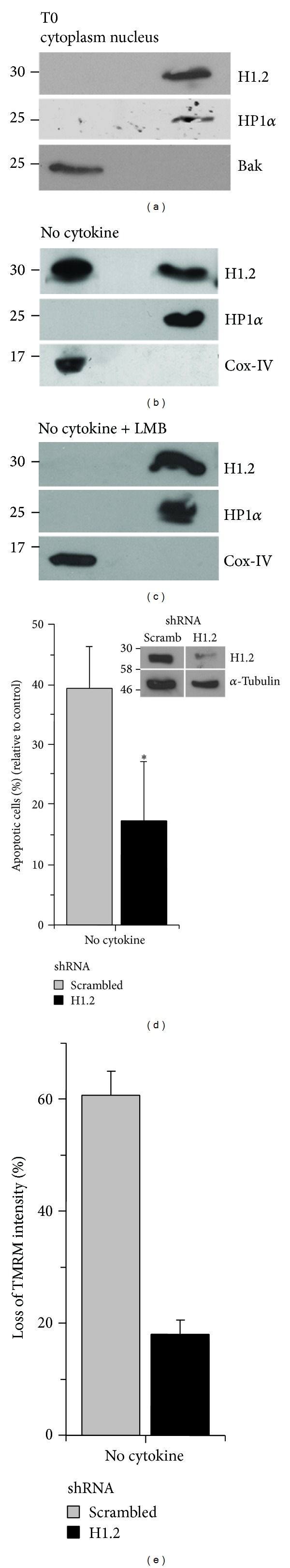
H1.2 is an intermediate in the apoptotic cascade triggered by cytokine deprivation. (a)–(c) 10 × 10^6^ T-effectors were cultured in the indicated conditions (T0: freshly activated) and 6 hours later subject to subcellular fractionation. Immunoblots of nuclear and cytoplasmic fractions were probed for H1.2, HP1*α*, Bak, or Cox-IV. Data are representative of three independent trials. (d)-(e) T-effectors transduced using retrovirus expressing scrambled control or histone H1.2 shRNA, as described in Materials and Methods, were cultured with or without cytokine for 10 hours. Cells were scored for apoptotic nuclear damage using Hoechst-33342 (d) or mitochondrial transmembrane potential using TMRM (e) as described in Materials and Methods. Graphs show mean ± SD from 5 experiments in (d) and 2 experiments in (e), normalized to cultures with cytokine. Inset: Immunoblot for H1.2 protein and tubulin (parity in loading) in shRNA expressing T-effectors. *P < 0.01.

H1.2 is typically detected in the nucleus as evidenced in the analysis of nuclear and cytoplasmic fractions of cells cultured in conditions that promote survival (Figure 2(a)). In contrast to this distribution, subcellular fractionation of cells following cytokine withdrawal (within 6 hours of the deprivation protocol) was characterized by the appearance of cytoplasmic pools of H1.2 (Figure 2(b)). This positions the translocation of H1.2 at a relatively early time-point in the apoptotic process, well before overt evidence of nuclear damage in experimental conditions. The distribution of the mitochondrial resident protein Cox-IV and the nuclear protein HP1*α* established purity of the cytoplasmic and nuclear fractions, respectively, in these assays. Consistent with the inhibition of apoptotic nuclear damage, if LMB was included in cytokine-withdrawal conditions, the distribution of H1.2 was restored to the pattern of live cells and only detected in the nucleus (Figure 2(c)). To assess the functional implications of the change in H1.2 localization and elucidate its role, if any, in apoptosis, RNA interference approaches were employed to ablate H1.2 function in T-effectors.

T-effectors were transduced using retroviruses with shRNA to H1.2 or a scrambled control and cultured in puromycin containing medium, as described in materials and Methods, in order to enrich for transfected cells [17]. Efficacy of the shRNA for depletion of H1.2 was established in the NIH3T3 fibroblast cell line (not shown) and as shown demonstrably reduced protein expression in T-effectors (Figure 2(d), inset). T-effectors treated with scrambled or H1.2 shRNA were assessed for their response to cytokine deprivation in the experiments that follow. In contrast to cells transduced with scrambled shRNA, cells with reduced H1.2 protein levels were protected from nuclear damage triggered by cytokine deprivation (Figure 2(d)). In order to assess if loss of H1.2 also protected from mitochondrial damage we used flow cytometry based analysis of intact cells. Loss of mitochondrial transmembrane potential is a sensitive indicator of damage to the organelle and characteristic of cells undergoing apoptosis. Hence we tested if H1.2 ablation protected T-effectors from mitochondrial damage triggered by cytokine deprivation. In these experiments, changes in mitochondrial activity were assessed by the uptake of the potentiometric dye TMRM. In contrast to cells expressing the control, scrambled shRNA, TMRM intensity was comparable in the presence or absence of cytokine in cells with an ablation of H1.2 consistent with protection from death (Figure 2(e)). This strongly suggests that reduced H1.2 levels protected cells from damage to mitochondria otherwise observed in cells in conditions of cytokine withdrawal. We did not observe deleterious effects of H1.2 depletion in T-effectors continued in culture in cytokine, indicating a specific role for H1.2 in the response to apoptotic stimuli. These data positioned H1.2 translocation and activity at a step controlling events culminating in compromised mitochondrial function and nuclear integrity in the apoptotic cascades in T-effectors.

### 3.3. H1.2 Associates with Bak in T-Effectors

**Figure 3:**
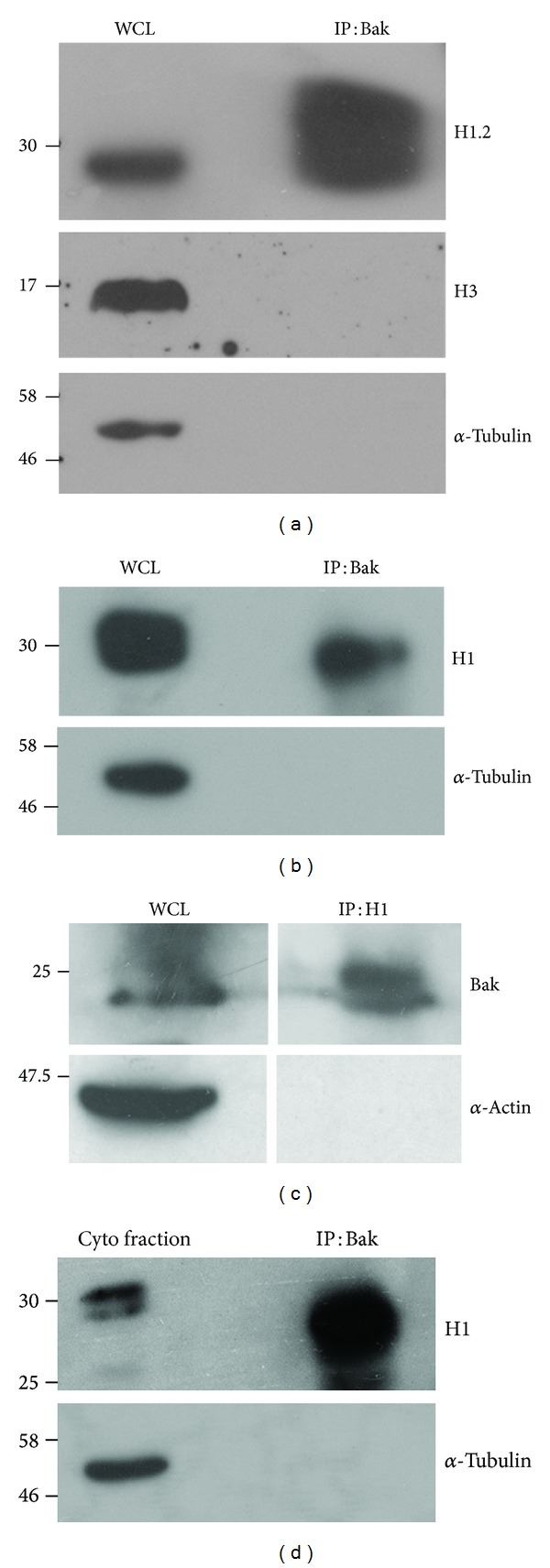
H1.2 immunoprecipitates with the mitochondrial outer-membrane resident Bak in T-effectors. (a)-(b) 10 × 10^6^ T-effectors were cultured without cytokine and 6 hours later immunoprecipitated (IP) with an antibody to Bak. Immunoblots of whole cell lysates (WCL) or the IP were probed with an antibody to H1.2 (a) or a pan H1 antibody (b) and *α*-Tubulin. (c) Reverse IP with an antibody to H1 in T-effectors cultured as described in (a)-(b) and the immunoblot probed for Bak. Actin is the specificity control. (d) 10 × 10^6^ T-effectors were cultured without cytokine for 6 hours and the cytoplasmic fraction obtained by subcellular fractionation as described in Materials and Methods was used as input for an IP with an antibody to Bak. A representative immunoblot for H1 in the complex IP is shown. *α*-Tubulin is the specificity control.

H1.2 has been reported to localize to the mitochondrion and participate in apoptotic cascades involving the mitochondrial resident protein Bak [25, 26]. The experiments described in the preceding sections indicated that H1.2 activity converged on mitochondrial function. Hence, we next tested for interactions between Bak and H1.2 in T-effectors under cytokine deprivation. The immune-precipitation (IP) of these proteins as a complex is evidence of a physical association (Figure 3(a)), which was confirmed using antibodies that recognize the H1 family of proteins or with an antibody specific to H1.2 (Figures 3(a) and 3(b)). Although the lysates used as the input for IP analysis are a postnuclear supernatant, we confirmed that core histone H3 was not detected in the immunoprecipitated complex (IP) indicating that nuclear pools of H1.2 were unlikely to be detected in the immunoblots (Figure 3(a)). Mindful of the detergent sensitivities of Bcl-2 family proteins [27], these experiments used the milder detergent CHAPS to generate cell lysates to assess these interactions. The association between Bak and H1.2 in T-cells was also confirmed by reverse immunoprecipitation (Figure 3(c)). In order to rule out possible contamination of the immunoprecipitated complex with nuclear proteins, the cytoplasmic fraction of T-effectors in deprivation (purity ascertained by the exclusion of the nuclear protein HP1*α*) was used to immunoprecipitate Bak. H1.2 immunoprecipitated in the complex with Bak when the cytoplasmic fraction was used as input as well, confirming that the association is occurring outside the nucleus (Figure 3(d)). The experiments thus far implicate H1.2 as key intermediate in the apoptotic response of T-effectors. In previous work [17], hierarchical interactions involving the Jun-N-terminal kinase (JNK) have been demonstrated to regulate T-effector apoptosis. Therefore, in the experiments that follow we attempted to position H1.2 activity in the context of known premitochondrial intermediates in the apoptotic cascade in T-effectors.

### 3.4. JNK Activity Regulates H1.2 Dynamics in T-Effectors

**Figure 4:**
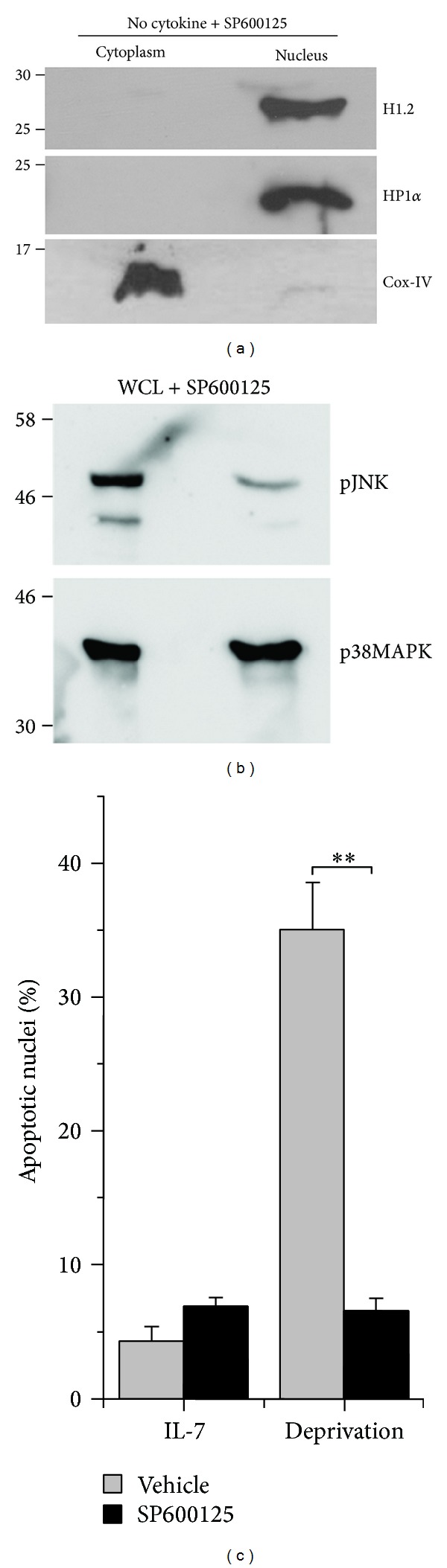
JNK regulates H1.2 displacement in T-effectors. (a) Representative immunoblot of nuclear and cytoplasmic fractions of T-effectors cultured without cytokine +100 nM SP600125 for 6 hours. The immunoblots were probed with antibodies to H1.2, HP1*α*, and Cox-IV and controlled with fractions generated in previous experiments. Data shown is representative of two independent trials. (b) Representative immunoblot for phosphorylated JNK in T-effectors cultured as described in (a). p38MAPK is the loading control. (c) Apoptotic nuclear damage in T-effectors cultured with and without cytokine (IL-7) in the presence or absence of vehicle control or 100 nM SP600125 for 24 hours. The graph shows the mean ± SD from 3 experiments. **P < 0.001.

ROS-dependent activation of the JNK regulates deprivation-induced apoptosis in T-effectors and apoptotic cascades in other cell types [17, 28]. Hence we asked if JNK inhibited the translocation of H1.2 in T-effectors. To modulate JNK activity we used the inhibitor, SP610025, demonstrated in earlier experiments to regulate JNK activity in T-effectors [17]. In the analysis of nuclear and cytoplasmic fractions, in cultures where SP610025 was added at the onset of the deprivation protocol, the distribution of H1.2 was comparable to control cells and detected only in the nuclear fraction (Figure 4(a)). Confirming earlier reports and consistent with its effect on the subcellular localization of H1.2, blocking JNK (Figure 4(b)) also protected T-cells from apoptotic damage (Figure 4(c)). Growing evidence of cross talk between signal transduction pathways and the metabolic status of cells, wherein evidence that metabolic stress triggers changes in acetylation and consequently mitochondrial function [29–31], prompted us to assess possible regulation of T-effector apoptosis by cellular acetylation machinery.

### 3.5. Acetylation Dependence of T-Effector Apoptosis

**Figure 5:**
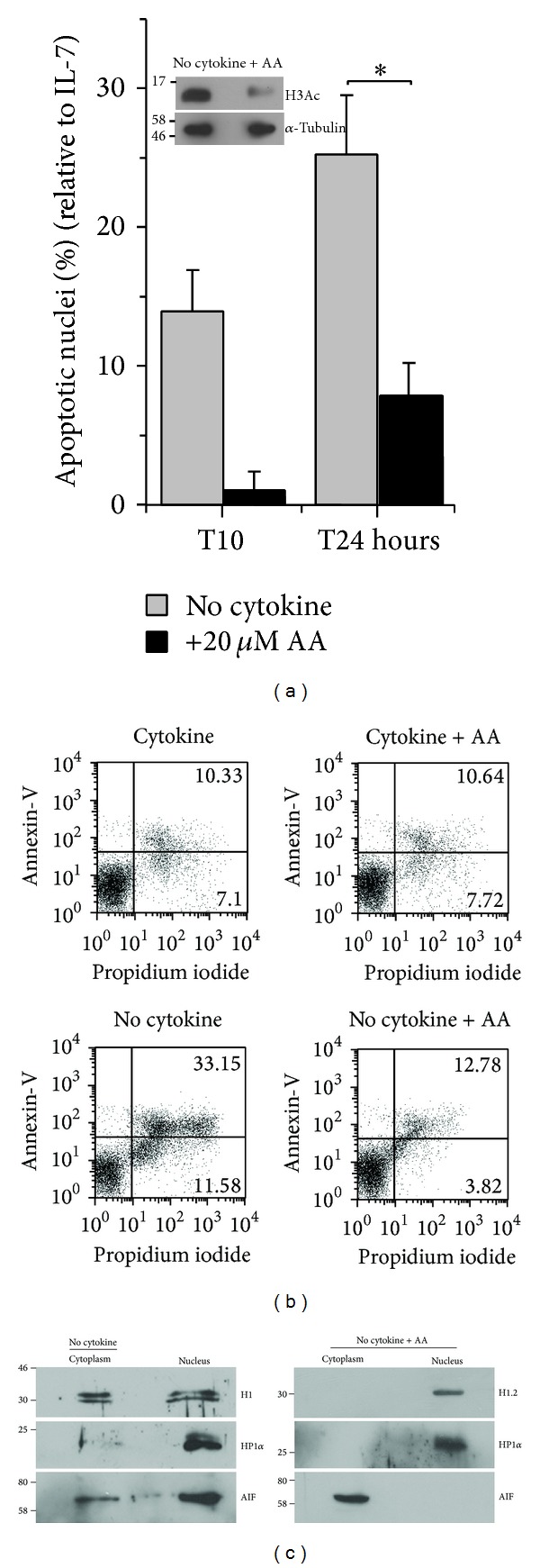
Acetylation dependence of the sub-cellular localization of H1.2 and apoptosis in T-effectors. (a) Apoptotic nuclear damage in T-effectors cultured without cytokine IL-7 (20 ng/ml) and with or without anacardic acid (AA) (20 *μ*M) for indicated time points. The graph shows the mean ± SD from three experiments. Inset: immunoblot for acetylated H3 (H3Ac) and *α*-tubulin in T-effectors cultured without cytokine (for 6 hours) in the presence or absence of AA. (b) T-effectors cultured for 18 hours in the indicated conditions were stained with propidium iodide (*x*-axis) and Annexin-V (*y*-axis) analyzed by flow cytometry. Values in the upper right quadrant, in each plot, indicate dead cells. (c) 10 × 10^6^ T-effectors were cultured without cytokine IL-7 (20 ng/ml) and with or without AA (20 *μ*M) for 6 hours. Immunoblots of nuclear and cytoplasmic fractions were probed for H1 or H1.2, HP1*α*, and AIF. Data shown is representative of three independent trials.

Mounting evidence of proteins modified by acetylation outside of conventional chromatin targets suggest a broader array and range of cellular processes regulated by this modification [32–34]. As a first approximation, a possible role of acetylation in the activation of the H1.2 mediated apoptotic cascade in T-effectors was tested. Anacardic acid (AA), a broad-spectrum inhibitor of lysine acetyl-transferases protected T-cells from apoptotic damage as seen in assays of nuclear morphology (Figure 5(a)). To assess if AA delayed rather than inhibited cell death, the uptake of propidium iodide (PI) was also measured in these conditions. After 18 hours in culture without cytokine, the coincident staining with PI and Annexin-V indicated the occurrence of membrane damage, which is in contrast to cells cultured in cytokine (Figure 5(b)). In cells that were cultured with AA, PI and Annexin-V staining was comparable (and low), indicating that AA prevented the loss of membrane integrity (Figure 5(b)). Further, the translocation of H1.2 (and AIF) was inhibited in cells treated with AA relative to the group cultured without cytokine (Figure 5(c)), supporting a possible role for changes in acetylation states as a regulatory step in the apoptotic response in T-effectors. The molecular target(s) of this modification remains to be identified. Experiments discriminating between the possibilities that H1.2 displacement may be a response to acetylation-dependent changes of chromatin organization vis-à-vis effects on histones or by the modulation of nonhistone proteins would provide additional insight into this regulation.

## 4. Discussion

Competition for limiting nutrients in the extracellular milieu is a widely prevalent mechanism for the regulation of cell number and mechanisms by which cells perceive these changes in their microenvironment continue to be actively investigated. Reduced availability of cell extrinsic growth factors frequently underlies the coordinated deletion of cells overproduced during development or differentiation. We address this question in a model system that recapitulates the deletion (induced by cytokine withdrawal) of differentiated T-cells in cell culture. We provide evidence that the nucleus functions as a site for integration of extracellular nutritional cues with significant consequences to T-cell survival. This was indicated in the experiments wherein LMB blocked apoptotic damage in T-effectors in response to cytokine deprivation implicating a nuclear intermediate in the signaling cascade. Involvement of nuclear events was confirmed in experiments where, following cytokine withdrawal, we observed that H1.2 was displaced from the nucleus to translocate to the mitochondrion, initiating cascades culminating in cell death. H1.2 translocation from the nucleus has been previously reported in immature thymocytes and chronic lymphocytic leukemic cells in response to genotoxic and nongenotoxic drug treatment [25, 26, 35]. In support of this, we present evidence for H1.2 interactions with the mitochondrial outer-membrane resident, the proapoptotic protein Bak, and its regulation of ensuing damage to mitochondria and the nucleus in T-effectors undergoing apoptosis. These data open up possibilities—not mutually exclusive—of H1.2 associated molecules that regulate apoptotic outcomes or, modifications to H1.2 itself that may be necessary for its activity at the mitochondrion.

A hierarchy of interactions involving Bcl-2 family proteins (Bax and Bak amongst others) and reactive oxygen species are implicated in the apoptotic deletion of T-cells [3, 13–15, 17, 18]. Current understanding of apoptotic signaling positioned molecules with well-characterized functions at the mitochondrion as key intermediates in T-cell apoptosis. Since ablation of H1.2 protected cells from apoptosis in culture, the data suggests an intriguing possibility of amplification or reinforcing loop mediated by H1.2, which converges on the mitochondrion to propagate the apoptotic cascade. We speculated that if signaling resulting from H1.2 and ROS activation converges on a single event/organelle, common regulatory processes might be expected. Earlier work from the laboratory had shown that Jun-N-terminal kinase (JNK) was activated by NADPH-mediated ROS production and regulated mitochondrial damage in the apoptotic response of T-effectors [17]. Supporting the possibility of a hierarchy of interactions, JNK activity controlled the subcellular distribution of H1.2, that is, its liberation from the nucleus following growth factor deprivation. The underlying molecular features and consequences of H1.2 association with Bak remain to be elucidated. Based on the observations in this study we speculate that chromatin may execute a nontranscriptional response to JNK activity to initiate the observed nonnuclear functions of H1.2. This outcome may be linked to the modulation of epigenetic states by JNK activity, which has precedence in other systems [36–38].

The protective effect of AA is consistent with a role for acetylation in the regulation of H1.2 relocation and consequent apoptosis. The possibility that lysine acetyl-transferases form an instructive element in the apoptotic H1.2-dependent cascade suggested by these observations, however, requires more detailed analysis. Our attempts to track T-cell contraction following antigen challenge in mice injected with AA were unsuccessful because of the inhibitory effects of AA on T-cell activation responses *in vivo* (not shown). Genetic manipulations of H1.2 levels in T-cell populations are underway to directly envisage the role of H1.2 pathway in promoting survival of T effectors *in vivo*.

Taken together, the experiments suggest that cytokine inputs necessary for nutrient uptake and consequently survival in T-effectors are integrated in the nucleus. These observations position H1.2 in the molecular coupling of genomic integrity and mitochondrial function, demonstrated here in the programmed deletion of T-effectors. It is tempting to speculate that, since cellular responses to metabolic stressors are conserved across cell types, the pathway described here is likely activated in other cellular contexts involving homeostasis of differentiated cells.

## 5. Conclusions

Competition for limiting nutrients in the extracellular milieu is a ubiquitous mechanism for the regulation of cell number. Intracellular signaling networks constituting the cellular response to these changes remain an active area of investigation. In this study we provide evidence that events in the nucleus integrate extracellular nutritional cues in T-effector populations and these events are intricately connected to the apoptotic response elicited by nutritional deprivation. Specifically, in response to cytokine withdrawal the linker histone H1.2 is displaced from the nucleus to translocate to the mitochondrion and associate with the mitochondrial resident protein Bak to trigger T-effector apoptosis. Finally, H1.2 dynamics are regulated by JNK signaling and responsive to changes in the acetylation status in T-cells.

## References

[1] Hengartner MO (2000). The biochemistry of apoptosis. *Nature*.

[2] Ashkenazi A, Dixit VM (1998). Death receptors: signaling and modulation. *Science*.

[3] Rathmell JC, Lindsten T, Zong WX, Cinalli RM, Thompson CB (2002). Deficiency in Bak and Bax perturbs thymic selection and lymphoid homeostasis. *Nature Immunology*.

[4] Youle RJ, Strasser A (2008). The BCL-2 protein family: opposing activities that mediate cell death. *Nature Reviews Molecular Cell Biology*.

[5] Aebischer J, Sturny R, Andrieu D (2011). Necdin protects embryonic motoneurons from programmed cell death. *PLoS ONE*.

[6] DiNapoli L, Batchvarov J, Capel B (2006). FGF9 promotes survival of germ cells in the fetal testis. *Development*.

[7] Goldrath AW, Bevan MJ (1999). Selecting and maintaining a diverse T-cell repertoire. *Nature*.

[8] Yuan J, Yankner BA (2000). Apoptosis in the nervous system. *Nature*.

[9] Barker V, Middleton G, Davey F, Davies AM (2001). TNF*α* contributes to the death of NGF-dependent neurons during development. *Nature Neuroscience*.

[10] Rathmell JC, Heiden MGV, Harris MH, Frauwirth KA, Thompson CB (2000). In the absence of extrinsic signals, nutrient utilization by lymphocytes is insufficient to maintain either cell size or viability. *Molecular Cell*.

[11] Tan JT, Dudl E, LeRoy E (2001). IL-7 is critical for homeostatic proliferation and survival of naïve T cells. *Proceedings of the National Academy of Sciences of the United States of America*.

[12] Lenardo M, Chan FK, Hornung F (1999). Mature T lymphocyte apoptosis—immune regulation in a dynamic and unpredictable antigenic environment. *Annual Review of Immunology*.

[13] Fischer SF, Belz GT, Strasser A (2008). BH3-only protein Puma contributes to death of antigen-specific T cells during shutdown of an immune response to acute viral infection. *Proceedings of the National Academy of Sciences of the United States of America*.

[14] Strasser A, Pellegrini M (2004). T-lymphocyte death during shutdown of an immune response. *Trends in Immunology*.

[15] Pellegrini M, Belz G, Bouillet P, Strasser A (2003). Shutdown of an acute T cell immune response to viral infection is mediated by the proapoptotic Bcl-2 homology 3-only protein Bim. *Proceedings of the National Academy of Sciences of the United States of America*.

[16] Vella AT, Dow S, Potter TA, Kappler J, Marrack P (1998). Cytokine-induced survival of activated T cells in vitro and in vivo. *Proceedings of the National Academy of Sciences of the United States of America*.

[17] Purushothaman D, Sarin A (2009). Cytokine-dependent regulation of NADPH oxidase activity and the consequences for activated T cell homeostasis. *Journal of Experimental Medicine*.

[18] Hildeman DA, Mitchell T, Teague TK (1999). Reactive oxygen species regulate activation-induced T cell apoptosis. *Immunity*.

[19] Gupta S, Marcel N, Talwar S (2012). Developmental heterogeneity in DNA packaging patterns influences T-cell activation and transmigration. *PLoS ONE*.

[20] Perumalsamy LR, Marcel N, Kulkarni S, Radtke F, Sarin A (2012). Distinct spatial and molecular features of notch pathway assembly in regulatory T cells. *Science Signaling*.

[21] Purushothaman D, Marcel N, Garg M, Venkataraman R, Sarin A (2013). Apoptotic programs are determined during lineage commitment of CD4+ T effectors: selective regulation of T effector-memory apoptosis by inducible nitric oxide synthase. *Journal of Immunology*.

[22] Lorenzo HK, Susin SA, Penninger J, Kroemer G (1999). Apoptosis inducing factor (AIF): a phylogenetically old, caspase-independent effector of cell death. *Cell Death and Differentiation*.

[23] Srivastava S, Banerjee H, Chaudhry A (2007). Apoptosis-inducing factor regulates death in peripheral T cells. *Journal of Immunology*.

[24] Abkallo HM, Kawano H, Watanabe K, Kobayashi NA (2011). New cell-based reporter system for sensitive screening of nuclear export inhibitors. *Drug Discoveries and Therapeutics*.

[25] Konishi A, Shimizu S, Hirota J (2003). Involvement of histone H1.2 in apoptosis induced by DNA double-strand breaks. *Cell*.

[26] Okamura H, Yoshida K, Amorim BR, Haneji T (2008). Histone H1.2 is translocated to mitochondria and associates with bak in bleomycin-induced apoptotic cells. *Journal of Cellular Biochemistry*.

[27] Hsu YT, Youle RJ (1997). Nonionic detergents induce dimerization among members of the Bcl-2 family. *The Journal of Biological Chemistry*.

[28] Kamata H, Honda S, Maeda S, Chang L, Hirata H, Karin M (2005). Reactive oxygen species promote TNF*α*-induced death and sustained JNK activation by inhibiting MAP kinase phosphatases. *Cell*.

[29] Guan KL, Xiong Y (2011). Regulation of intermediary metabolism by protein acetylation. *Trends in Biochemical Sciences*.

[30] Wellen KE, Thompson CB (2012). A two-way street: reciprocal regulation of metabolism and signalling. *Nature Reviews Molecular Cell Biology*.

[31] Wellen KE, Hatzivassiliou G, Sachdeva UM, Bui TV, Cross JR, Thompson CB (2009). ATP-citrate lyase links cellular metabolism to histone acetylation. *Science*.

[32] Choudhary C, Kumar C, Gnad F (2009). Lysine acetylation targets protein complexes and co-regulates major cellular functions. *Science*.

[33] Kouzarides T (2000). Acetylation: a regulatory modification to rival phosphorylation. *EMBO Journal*.

[34] Spange S, Wagner T, Heinzel T, Krämer OH (2009). Acetylation of non-histone proteins modulates cellular signalling at multiple levels. *International Journal of Biochemistry and Cell Biology*.

[35] Giné E, Crespo M, Muntañola A (2008). Induction of histone H1.2 cytosolic release in chronic lymphocytic leukemia cells after genotoxic and non-genotoxic treatment. *Haematologica*.

[36] Tiwari VK, Stadler MB, Wirbelauer C, Paro R, Schübeler D, Beisel C (2012). A chromatin-modifying function of JNK during stem cell differentiation. *Nature Genetics*.

[37] Wu J, Zhang X, Nauta HJ, Lin Q, Li J, Fang L (2008). JNK1 regulates histone acetylation in trigeminal neurons following chemical stimulation. *Biochemical and Biophysical Research Communications*.

[38] Sundaresan NR, Vasudevan P, Zhong L (2012). The sirtuin SIRT6 blocks IGF-Akt signaling and development of cardiac hypertrophy by targeting c-Jun. *Nature Medicine*.

